# Protocol for the purification of replisomes from the *Xenopus laevis* egg extract system for single-particle cryo-EM analysis

**DOI:** 10.1016/j.xpro.2024.103237

**Published:** 2024-08-08

**Authors:** Paolo Passaretti, Milos A. Cvetkovic, Alessandro Costa, Agnieszka Gambus

**Affiliations:** 1 Institute of Cancer and Genomic Sciences, Birmingham Centre for Genome Biology, University of Birmingham, B15 2TT Birmingham, UK; 2 Macromolecular Machines Laboratory, The Francis Crick Institute, NW1 1AT London, UK

**Keywords:** Model Organisms, Protein expression and purification, Cryo-EM

## Abstract

Here, we present a large-scale FLAG immunoprecipitation protocol to isolate large protein complexes driving DNA replication at replicating chromatin assembled in *Xenopus laevis* egg extract. We describe how to prepare demembranated sperm nuclei (DNA) and low-speed supernatant egg extract (LSS) and present detailed procedures for sample preparation and application onto grids for negative stain electron microscopy (NS-EM) and cryoelectron microscopy (cryo-EM).

For complete details on the use and execution of this protocol, please refer to Cvetkovic et al.^1^

## Highlights

• Steps for preparing demembranated sperm nuclei to use with *Xenopus* egg extract
• Protocol for preparing *Xenopus laevis* low-speed supernatant (LSS) egg extract
• Steps to isolate replisomes via large-scale IP from replicating LSS
• Procedures to deposit the sample on EM grids for NS-EM and cryo-EM

## Abstract (editor highlights)

Publisher’s note: Undertaking any experimental protocol requires adherence to local institutional guidelines for laboratory safety and ethics.

## Graphical abstract

**Figure:**
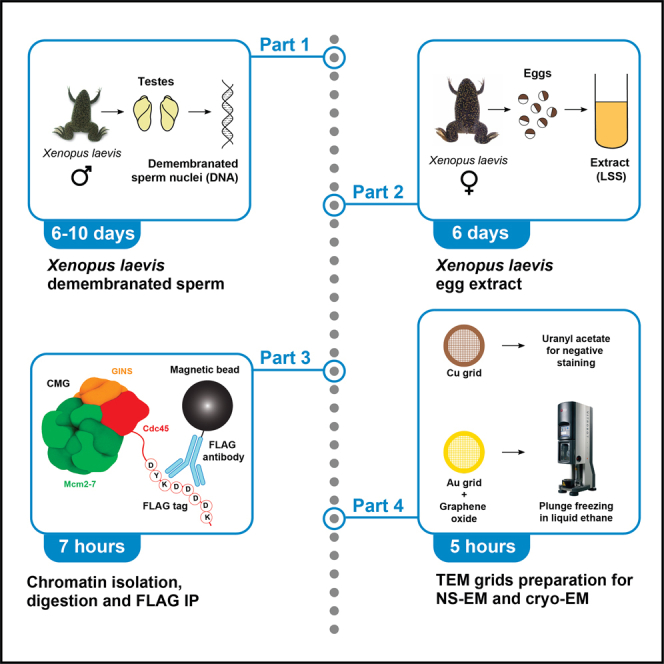

## Before you begin

### Animals

This procedure makes use of material generated by animals: African clawed toad (*Xenopus laevis*) and therefore requires access to a *Xenopus laevis* colony. All animal work requires appropriate ethical and legal approval and must be performed in accordance with legal standards. For preparation of egg extracts only fully grown females are used, while for preparation of demembranated sperm, only mature males are used.

**Figure 1:**
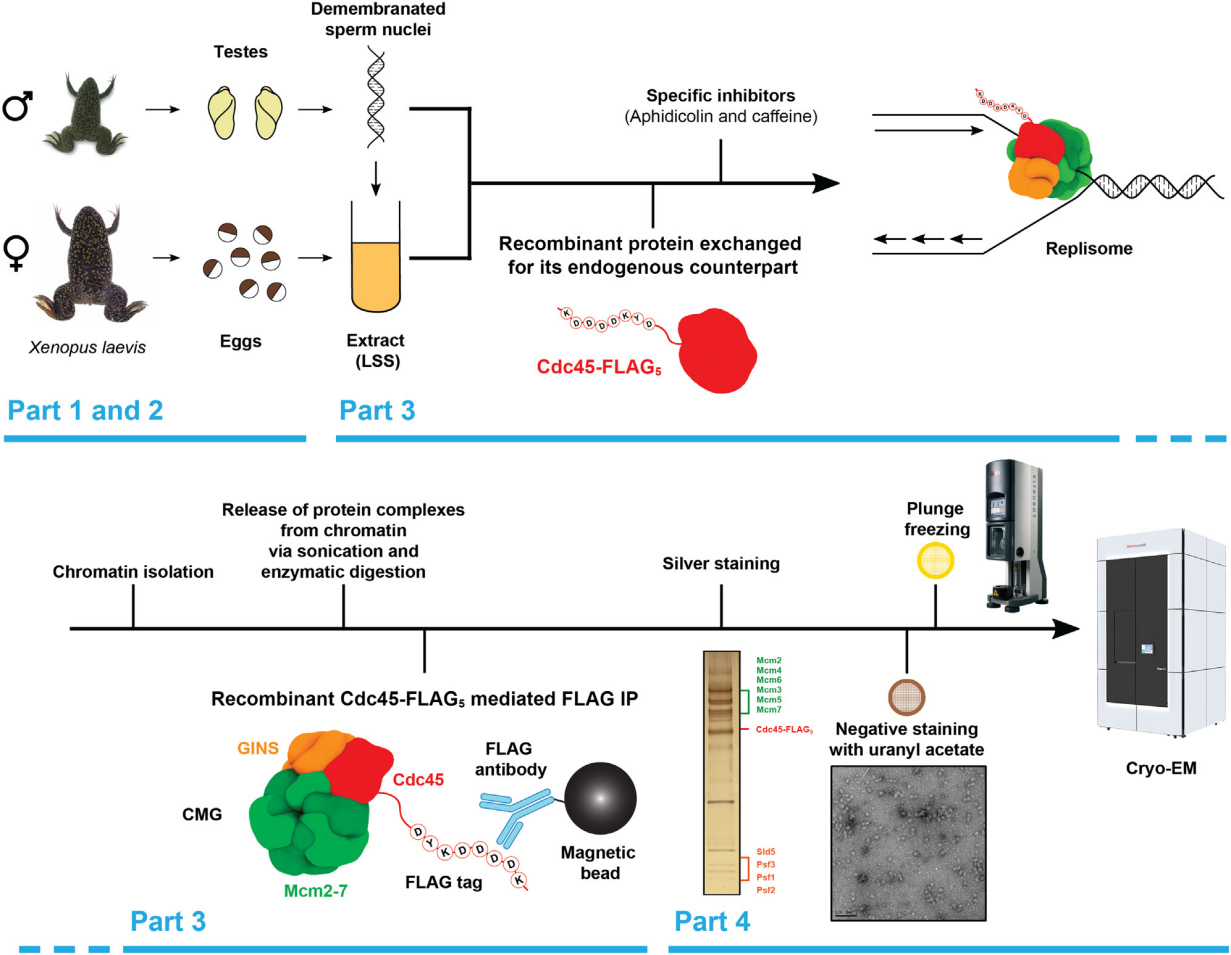
Schematic representation of the entire method pipeline In Part 1 and 2 we describe how to prepare *Xenopus laevis* demembranated sperm nuclei and Low-Speed Supernatant Egg Extract (LSS), respectively. Part 3 details how to perform the large-scale chromatin isolation and FLAG immunoprecipitation of replisomes. Finally, Part 4 describes how to evaluate sample quality via silver staining and negative staining EM, as well as how to prepare grids for cryo-EM image acquisition (Schematics and EM density from EMD-18195).^1^

*Xenopus laevis* egg extract was first developed as a model system over 40 years ago and ever since has enabled key discoveries in cell cycle research, especially in DNA replication, damage repair, and segregation fields.^2^ *Xenopus* eggs are full of accumulated cell cycle factors that are needed for completion of 12 rapid cleavage cell cycles at the beginning of frog embryo development. It is this high concentration of cell cycle factors that enables the extracts (liquid part of the egg) to support the cell cycle progression *in vitro.* The process of DNA replication in egg extract is executed and regulated in analogous way as *in vivo*. Xenopus eggs are arrested in metaphase of meiosis II and therefore to release the extract into interphase, that is compatible with further cell cycle progression, metaphase arrested egg extract is first supplemented with calcium to complete meiosis exit.^3^^,^^4^ Demembranated *Xenopus* sperm DNA (physiological substrate) added to interphase egg extract is first decondensed and assembled into chromatin and then it forms into interphase nuclei. Once nuclear assembly is complete, the DNA is efficiently and synchronously duplicated. Over the decades, different versions of egg extract preparation evolved to allow for specific analyses – for example nucleoplasmic extract (NPE) allows for replication of plasmid templates.^5^^,^^6^ For clarity and fullness of the methods, we describe here the protocols we use for demembranated sperm and low-speed supernatant (LSS) egg extract preparation, despite them being described previously elsewhere (Figure 1).^7^^,^^8^

### Before starting *Xenopus laevis* demembranated sperm nuclei preparation

**Timing: 6–10 days (total)**

**Timing: 1 h (for step 2)**

**Figure 2:**
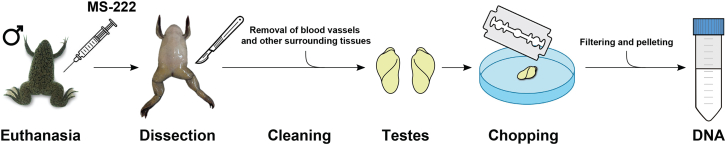
Schematic representation of the major steps of demembranated sperm nuclei preparation Frogs are euthanized, testes dissected and cleaned. Subsequently, testes are finely chopped with a razor blade, filtered, aliquoted and stored appropriately.

This step details the extraction and processing of frog testes to procure demembranated sperm nuclei, which is the template DNA for DNA replication within *Xenopus laevis* egg extract systems. The protocol initiates with induction of sperm maturation in male frogs, followed by their humane euthanization and careful dissection of their testes. These organs undergo meticulous cleaning and a series of treatments involving chopping, filtering, and centrifugation to eliminate membranes and other cellular components, ultimately liberating sperm nuclei. Subsequently, the sperm nuclei undergo demembranation, are quantified using microscopy, and are then stored in aliquots for future utilization in conjunction with egg extract (Figure 2).

1. Folligon injection and euthanasia of frog males (Point 1 and 2).
  a. Folligon and MS-222 are prepared fresh immediately before using.
2. Before starting testes dissection (Point 3).
  a. Have ready on ice in a bucket:
    i. EB.
    ii. EB-glycerol.
    iii. SuNaSp.
    iv. SuNaSp-BSA.
    v. Lysolecithin.
    vi. SYTOX Orange.
  b. Switch on Megafuge 16 with TX-400 swinging bucket rotor and set the temperature at 4°C.

### Before starting *Xenopus laevis* Low-Speed Supernatant Egg Extract preparation

**Timing: 6 days (total)**

**Timing: 30 min (for step 3)**

**Timing: 1 h (for step 4)**

**Timing: 1 h (for step 5)**

**Figure 3:**
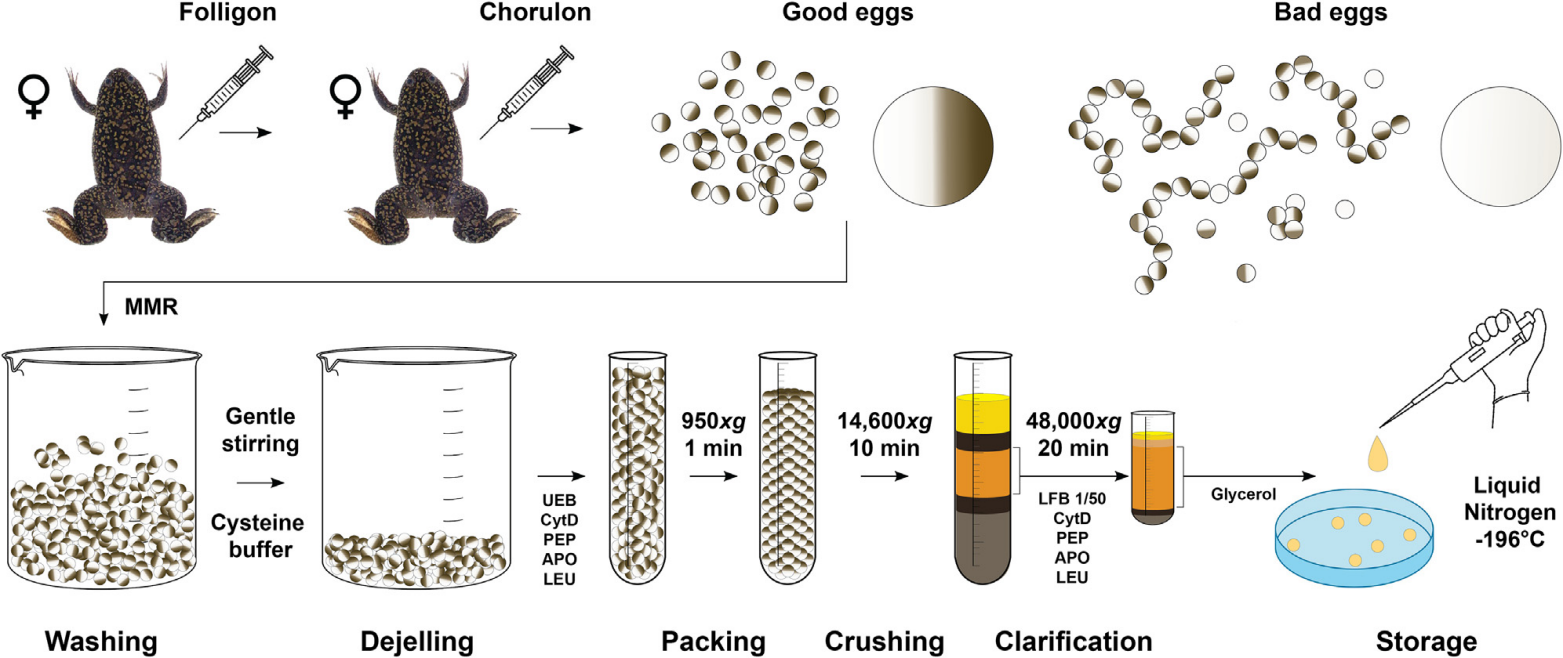
Schematic representation of the major steps of LSS preparation After injection of Folligon and Chorulon, frogs will lay eggs. Bad quality eggs are discarded together with any debris (feces, shed skin). Eggs are extensively washed, dejellied and apoptotic eggs removed with a plastic Pasteur pipette. Clean eggs are aliquoted into tubes, packed and spin crushed. The central layer is collected using a syringe and clarified via ultracentrifugation. Finally, the top layer containing fats is discarded and the extract collected from the top, making sure that the bottom dark layer is not perturbed at any time.

This process outlines the steps involved in preparation of egg extract. The female frogs are stimulated to mature more oocytes and then to lay eggs. The collected eggs undergo a series of procedures, including cleaning, centrifugal packing/crushing, and extraction of the cytosolic layer. This resulting substance is known as Low-Speed Supernatant Egg Extract (LSS), capable of supporting a single round of DNA replication upon the introduction of demembranated sperm nuclei (DNA) and various activators (Figure 3).

3. Day 1 (Point 6).
  a. Prepare fresh Folligon immediately before injection.
4. Day 2, before frog injections (Point 7).
  a. Prepare:
    i. 10x MMR, keep it at 20°C–25°C.
    ii. 1x MMR, store overnight at 4°C.
    iii. UEB, store overnight at 4°C.
  b. Place the ultracentrifuge rotor and the swinging tubes adaptors (SW50.1) in the cold room to have them ready at 4°C on Day 3 (Point 8).
  c. Prepare fresh Chorulon immediately before injection.
5. Day 3, before collecting eggs (Point 8).
  a. Prepare:
    i. Cysteine solution.
    ii. Glycerol 50%.
  b. Thaw and keep on ice in a bucket:
    i. APR.
    ii. LEU.
    iii. PEP.
    iv. CytD.
    v. LFB 1/50.
  c. Switch on the High-speed centrifuge, Avanti J-E with JS-13.1 swinging bucket rotor and set the temperature at 20°C.
  d. Switch on the Ultra-centrifuge, Optima LE-80K with SW50.1 swinging bucket rotor and set the temperature at 4°C.
  e. Collect liquid nitrogen.
  f. Arrange all the other required tools available near your working area.
    i. 20-G needles.
    ii. Syringes 1 mL.
    iii. Round bottom centrifuge tubes 14 mL.
    iv. Plastic Pasteur pipette.
    v. Plastic Petri dishes of 9 cm diameter.

### Before starting large scale chromatin isolation and FLAG immunoprecipitation

**Timing: 7 h (total)**

**Timing: 15 min (for step 6)**

This protocol includes the detailed methodology to set up a DNA replication reaction with LSS in the presence of aphidicolin and caffeine. The role of caffeine is to inhibit ATM/ATR checkpoint kinases and boost the origin firing, while aphidicolin inhibits family B polymerases, inducing their decoupling from the CMG helicase. This results in an enrichment of stalled replisomes on chromatin. Moreover, recombinant Cdc45-FLAG_5_ (Addgene #208796) is introduced to outcompete endogenous Cdc45, allowing the purification of these replisomes via FLAG immunoprecipitation with magnetic beads functionalized with anti-FLAG antibodies.

6. Before starting the large-scale FLAG immunoprecipitation (FLAG-IP) from chromatin (Point 10).
  a. Prepare:
    i. 2-chloroacetamide, keep it at 20°C–25°C.
    ii. Caffeine, keep it at 20°C–25°C.
    iii. ANIB, keep it on ice.
    iv. ANIB-sucrose, keep it on ice.
  b. Thaw and keep on ice in a bucket:
    i. ER.
    ii. CHX.
    iii. CaCl_2_.
    iv. sperm DNA.
    v. Cdc45-FLAG_5_ (More details about the protein purification can be found in the method section of^1^).
    vi. Aphidicolin.
  c. Switch on Megafuge 16 with TX-400 swinging bucket rotor and set the temperature at 4°C.

### Before starting analysis of purified replisomes

**Timing: 1 day (total)**

**Timing: 10 min (for step 7)**

**Timing: 1 h (for step 8)**

The material isolated by the FLAG-IP in Part 3 can next be analyzed for purity, concentration, and composition by silver staining of an SDS-PAGE gel, by western blotting with appropriate antibodies, and by mass spectrometry. The samples of adequate concentration and purity can then be further analyzed for particle homogeneity, dispersion and concentration by negative staining and TEM. Finally, cryo-EM grids can be prepared.

***Note:*** The isolation of replisomes and preparation of the EM grids should be carried on in tandem on the same day to reduce loss of sample. Therefore, consider performing Part 3 and 4 on the same day.

7. Before starting sample preparation for negative stain electron microscopy (Point 16).
  a. If the uranyl acetate solution aliquots are kept at 20°C–25°C, place the aliquots on ice for 5–10 min before centrifugation.
  b. Cool the benchtop centrifuge to 4°C.
8. Before starting sample preparation for cryo-electron microscopy (Point 18).
  a. Prepare enough liquid nitrogen in a flask/Dewar for cooling and refilling the vitrification container set, and for the transfer of grids after plunge freezing.
  b. Preparation of graphene oxide grids is described in Point 17.
  c. Steps in setting up and cooling the vitrification container set, and in preparation of the Vitrobot are described in Point 18.

## Key resources table

**Table:** 

REAGENT or RESOURCE	SOURCE	IDENTIFIER
**Biological samples**		

*Xenopus laevis* low-speed supernatant egg extract (LSS)	This paper	N/A
*Xenopus laevis* demembranated sperm (DNA)	This paper	N/A

**Chemicals, peptides, and recombinant proteins**		

2-Chloroacetamide, ClCH₂CONH₂ (2-Ch)	Millipore	CAS: 79-07-2
Adenosine-5′-triphosphate (ATP)	Scientific Laboratory Supplies	CAS: 56-65-5
Anti-FLAG M2 magnetic beads	Sigma-Aldrich	Cat# M8823
Aphidicolin	Bio-Techne Limited	Cat# 5736/1
Aprotinin (APR)	Cambridge Bioscience	CAS: 9087-70-1
Benzonase nuclease	Sigma-Aldrich	E1014-25KU
Bovine serum albumin (BSA)	MP Biomedicals	CAS: 9048-46-8
Caffeine	Sigma-Aldrich	CAS: 58-08-2
Calcium chloride dihydrate, CaCl_2_ · 2 H_2_O (CaCl_2_)	Sigma-Aldrich	CAS: 10035-04-8
Cdc45-TEV-His_10_-FLAG_5_ (MW: 72.92 kDa; ε: 76,780 M^−1^ cm^−1^)	Cvetkovic et al.^1^	Addgene #208796
Chorulon human chorionic gonadotrophin (hCG)	MSD Animal Health	CAS: 23491-45-4
Creatine phosphate disodium salt	Sigma-Aldrich	CAS: 19333-65-4
Creatine phosphokinase	Sigma-Aldrich	CAS: 9001-15-4
Cycloheximide (CHX)	Millipore	CAS: 66-81-9
Cytochalasin D (CytD)	Sigma-Aldrich	CAS: 22144-77-0
Dimethyl sulfoxide (DMSO)	Sigma-Aldrich	CAS: 67-68-5
Dimethylformamide (DMF)	Sigma-Aldrich	CAS: 68-12-2
Dipotassium hydrogen phosphate, K_2_HPO_4_	Sigma-Aldrich	CAS: 7758-11-4
Dithiothreitol (DTT)	Promega	CAS: 3483-12-3
EDTA disodium salt dihydrate	Sigma-Aldrich	CAS: 60-00-4
EGTA	Sigma-Aldrich	CAS: 67-42-5
Folligon pregnant mare serum gonadotrophin (PMSG)	MSD Animal Health	CAS: 9002-70-4
Glycerol	Sigma-Aldrich	CAS: 56-81-5
Graphene oxide (GO)	Sigma-Aldrich	CAS: 1034343-98-0
HEPES	Fisher BioReagents	CAS: 7365-45-9
IGPAL CA-630	Sigma-Aldrich	CAS: 9002-93-1
L-cysteine	Sigma-Aldrich	CAS: 52-90-4
Leupeptin (LEU)	Cambridge Bioscience	CAS: 103476-89-7
Liquid ethane	N/A	CAS: 74-84-0
Liquid nitrogen	N/A	CAS: 7727-37-9
Loading buffer (LB) NuPAGE LDS sample buffer (4x)	Invitrogen	Cat# NP0007
Magnesium acetate, Mg(CH_3_COO)_2_ · 4 H_2_O (Mg(OAc)_2_)	Alfa Aesar	CAS: 16674-78-5
Magnesium chloride hexahydrate, MgCl_2_ · 6 H_2_O (MgCl_2_)	Fisher BioReagents	CAS: 7786-30-3
NuPAGE 4%–12%, Bis-Tris, 1.0–1.5 mm, Mini Protein Gels	Fisher Scientific	Cat# NP0323BOX
PageRuler prestained protein ladder, 10–180 kDa	Fisher Scientific	Cat# 26616
Pepstatin (PEP)	Cambridge Bioscience	CAS: 26305-03-3
Phenylmethylsulfonyl fluoride solution in ethanol (PMSF)	Sigma-Aldrich	CAS: 329-98-6
Potassium acetate, CH_3_COOK (KOAc)	Sigma-Aldrich	CAS: 127-08-2
Potassium phosphate, KH_2_PO_4_	Sigma-Aldrich	CAS: 7778-77-0
Proteinase K	Macherey-Nagel	Cat# 11912312
SimplyBlue SafeStain	Thermo Scientific	Cat# LC6060
Sodium bicarbonate (NaHCO_3_)	Sigma-Aldrich	CAS: 144-55-8
Sodium dodecyl sulfate (SDS)	Sigma-Aldrich	CAS: 151-21-3
Sodium orthovanadate (Na_3_VO_4_)	Fisher Scientific	CAS: 13721-39-6
Spermidine	Alfa Aesar	CAS: 124-20-9
Spermine	Sigma-Aldrich	CAS: 71-44-3
Sucrose	Sigma-Aldrich	CAS: 57-50-1
SYTOX Orange	Thermo Scientific	Cat# S11368
Tricaine methanesulfonate MS-222	Syndel	CAS: 886-86-2
Triple FLAG peptide (8.7 mM)	Stratech Scientific Ltd.	Cat# A6001
Triton X-100	Sigma-Aldrich	CAS: 9002-93-1
Tween 20	Sigma-Aldrich	CAS: 9005-64-5
Uranyl acetate (UA) powder	TAAB	CAS: 541-09-3
β-Glycerophosphate disodium salt pentahydrate	Alfa Aesar	CAS: 13408-09-8
β-Mercaptoethanol	Sigma-Aldrich	CAS: 60-24-2

**Critical commercial assays**		

SilverQuest silver staining kit	Invitrogen	Cat# LC6070

**Experimental models: Organisms/strains**		

Male and female *Xenopus laevis* frogs	Bred in-house, University of Birmingham Available to purchase from licensed breeders	N/A

**Other**		

20-G 0.9 × 25 mm needles	BD Microlance	N/A
25-G 0.5 × 16 mm needles	BD Microlance	N/A
Benchtop centrifuge, Megafuge 16 with TX-400 4 × 400 mL swinging bucket rotor with adaptors for 15/50 mL tubes.	Thermo Scientific	N/A
Benchtop microfuge Eppendorf 5417R with 8-place swinging bucket rotor	Eppendorf	N/A
Benchtop microfuge, Eppendorf 5418R with 18 × 1.5–2 mL fixed angle rotor	Eppendorf	N/A
Benchtop microfuge, Eppendorf 5424R with 24 × 1.5–2 mL fixed angle rotor	Eppendorf	N/A
Bioruptor standard sonication system	Diagenode	Cat# UCD-200
Blotting paper	Whatman	Cat# 1001-090
Carbon steel razor blades	Azpack	Cat# 11904325
ChemiDoc Touch imaging system	Bio-Rad	Cat# 1708370
Cold room 4°C	N/A	N/A
Dissection forceps	N/A	N/A
Forceps	N/A	N/A
Funnel	N/A	N/A
Glass slides and 0.5 cm diameter coverslips	N/A	N/A
Glow discharge system GloQube Plus	Quorum	N/A
Hemocytometer grid	N/A	N/A
High-speed centrifuge, Avanti J-E with JS-13.1 aluminum swinging-bucket rotor	Beckman Coulter	N/A
Immobilon-P PVDF membrane	Millipore	Cat# IPVH00010
Incubator 23°C, thermostatic cabinet	Lovibond	Cat# 2438200
Incubator 37°C, INCU-Line	VWR	N/A
Magnetic separation microtube rack, DynaMag-2 magnet	Thermo Fisher Scientific	Cat# 12321D
Mesh nylon filter 25 μm	NITEX	N/A
Metal spatula	N/A	N/A
Plastic Pasteur pipette	N/A	N/A
Plastic Petri dishes of 9 cm diameter	N/A	N/A
PowerPac basic power supply	Bio-Rad	Cat# 1645050
Rotary tube mixer with disc	Ratek	Cat# RSM7DC
Round-bottom centrifuge tubes 14 mL PP for eggs packing	Greiner Bio-One	Cat# 187261
Scalpels	N/A	
Syringes 1 and 5 mL	BD Plastipak	Cat# 303172, 309646
TEM grid for cryo-EM – UltrAuFoil R1.2/1.3, Au 300 mesh	Quantifoil Micro Tools GmbH	Cat# N1-A14nAu30-50
TEM grids for negative staining – EMResolutions C300Cu	EM Resolutions	Cat# C300Cu
Inverted microscope for fluorescence and transmitted light applications, e.g., EVOS XL core imaging system	Thermo Scientific	N/A
Tube roller SRT9	Stuart	Cat# 04653-04
Tweezers Style 4	Dumont	Cat# 0102-4-PO
Tweezers Style N5AC	Dumont	Cat# 0202-N5AC-PO
Ultra-centrifuge, Optima LE-80K with SW50.1 aluminum swinging bucket 6 × 5 mL rotor	Beckman Coulter	N/A
Ultracentrifuge 5 mL centrifuge tubes for eggs extract	Beckman Coulter	Cat# 326819
Vitrobot Mark IV System	Thermo Scientific	N/A

## Materials and equipment

**Table:** 

2-Chloroacetamide 500 mM	Dissolve 46.75 mg of 2-chloracetamide in 1 mL ddH_2_O. Prepare it fresh before adding to ANIB buffer and keep at 20°C–25°C. Caution: 2-Ch warrants careful handling as it may form combustible dust concentrations in the air, pose toxicity if ingested, trigger allergic skin reactions, and is suspected of harming fertility. Read SDS carefully.
Aphidicolin 8 mM	Dissolve 1 mg aphidicolin in 369.3 μL ddH_2_O, prepare 50 μL aliquots and store at −80°C. Maximum time for storage 1 year. Aphidicolin is a DNA polymerase α, δ and ε inhibitor and it exhibits selectivity over DNA polymerase β and γ.^9^
Aprotinin (APR) 10 mg/mL	Dissolve 10 mg aprotinin in 1 mL ddH_2_O, prepare 100 μL aliquots and store at −20°C or −80°C. Maximum time for storage 6 months. Aprotinin is a small protein which is an antifibrinolytic molecule that inhibits trypsin and related proteolytic enzymes.
ATP 100 mM	Dissolve 551 mg ATP in 8 mL ddH_2_O, adjust to pH 7.5 with NaOH and top up to 10 mL. Be careful, if the solution becomes too basic, ATP will hydrolyze. Prepare 10 μL aliquots and store at −80°C. Maximum time for storage 1 year.
BSA 30% (w/v)	Dissolve 300 mg in 1 mL ddH_2_O, prepare fresh when needed and keep it on ice.
CaCl_2_ 50 mM and 1 M	Dissolve 7.35 mg CaCl_2_ dihydrate in 1 mL ddH_2_O, filter with 0.22 μm filter, prepare 100 μL aliquots and store at −20°C or −80°C. Maximum time for storage 1 year. For 1M preparation, dissolve 73.5 g CaCl_2_ dihydrate in 500 mL ddH_2_O, filter with 0.22 μm filter and store at 20°C–25°C.
Caffeine 100 mM	Dissolve 19.41 mg caffeine in 1 mL ddH_2_O, prepare fresh when needed and keep at 20°C–25°C. It is light sensitive – keep protected from light. Caffeine inhibits ATM and ATR, promoting rapid and synchronous origin firing of DNA replication in the absence of DNA damage.^10^
Chorulon 1,000 IU/mL	Dissolve 1,500 IU of Chorulon in 1.5 mL of the provided solvent. Prepare it fresh before injecting and keep at 20°C–25°C. Load 550 μL Chorulon in 1 mL syringes with 25-G needle, remove any air from the syringe via ejecting 50 μL and inject 500 μL.
CHX 10 mg/mL	Dissolve 10 mg CHX in 1 mL ddH_2_O, prepare 100 μL aliquots and store at −20°C or −80°C. Maximum time for storage 1 year. Cycloheximide is a naturally occurring fungicide produced by the bacterium *Streptomyces griseus* that inhibits protein synthesis, thereby keeping the extract in the interphase of the cell cycle.
CytD 10 mg/mL	Dissolve 10 mg cytochalasin D in 1 mL ddH_2_O, prepare 50 μL aliquots and store at −20°C or −80°C. Maximum time for storage 1 year.
EDTA 0.5 M	Dissolve 186.1 g EDTA in 800 mL ddH_2_O, adjust to pH 8.0 with NaOH and top up to 1 L.
EGTA 0.5 M	Dissolve 190.2 g EGTA in 800 mL ddH_2_O, adjust to pH 8.0 with NaOH and top up to 1 L.
Folligon PMSG 1,000 IU/mL	Dissolve 5,000 IU of Folligon in 5 mL of the provided solvent. Prepare fresh before injecting and keep at 20°C–25°C. Load 200 μL Folligon in 1 mL syringes with 25-G needle, remove any air from the syringe via ejecting 50 μL and inject 150 μL.
Glycerol 50% (v/v)	Mix 50 mL glycerol with 50 mL ddH_2_O, filter with 0.22 μm filter and store at 20°C–25°C. Maximum time for storage 1 month.
HEPES-KOH 1 M pH 8	Dissolve 23.83 g HEPES in 100 mL ddH_2_O. Adjust to pH 8 with KOH before topping up the volume. Filter with 0.22 μm filter and store at −20°C. Maximum time for storage 6 months.
HEPES-KOH 50 mM and 1 M pH 7.6	Dissolve 1.19 g and 23.83 g HEPES in 100 mL ddH_2_O respectively. Adjust to pH 7.6 with KOH before topping up the volume. Filter with 0.22 μm filter and store at −20°C. Maximum time for storage 6 months.
KH_2_PO_4_ 0.5 M pH 8.0	Dissolve 34.02 g KH_2_PO_4_ with 500 mL of ddH_2_O to make 0.5 M KH_2_PO_4_ 0.5 M. Separately, dissolve 43.55 g K_2_HPO_4_ with 500 mL of ddH_2_O to make K_2_HPO_4_ 0.5 M. Add KH_2_PO_4_ to K_2_HPO_4_ until pH 8.0. Maximum time for storage 1 month.
Leupeptin (LEU) 10 mg/mL	Dissolve 10 mg leupeptin in 1 mL ddH_2_O, make 100 μL aliquots and store at −20°C or −80°C. Maximum time for storage 6 months. Leupeptin, also known as N-acetyl-L-leucyl-L-leucyl-L-argininal, is a naturally occurring protease inhibitor that can inhibit cysteine, serine, and threonine peptidases.
Lysolecithin 5 mg/mL	Dissolve 5 mg lysolecithin in 1 mL ddH_2_O, prepare 250 μL aliquots and store at −80°C. Maximum time for storage 6 months.
MMR 1x	Dilute 200 mL MMR 10x with 1800 mL ddH_2_O. This is prepared the day before needed and stored at 4°C.
Pepstatin (PEP) 10 mg/mL	Dissolve 10 mg pepstatin in 1 mL DMSO, prepare 100 μL aliquots and store at −20°C or −80°C. Maximum time for storage 1 year. Pepstatin is a hexa-peptide having the sequence Isovaleryl-Val-Val-Sta-Ala-Sta and is a potent inhibitor of aspartyl proteases.
Spermidine 500 mM	Dissolve 72.6 mg spermidine in 10 mL ddH_2_O, prepare 1 mL aliquots and store at −80°C. Maximum time for storage 1 year.
Spermine 500 mM	Dissolve 101.2 mg spermine in 10 mL ddH_2_O, prepare 1 mL aliquots and store at −80°C. Maximum time for storage 1 year.
Sucrose 40% (w/v)	Dissolve 20 g sucrose in 50 mL ddH_2_O, filter with 0.22 μm filter, and store at 4°C. Maximum time for storage 6 months.
SYTOX Orange 5 μM	Dilute 2 μL SYTOX Orange from the 5 mM purchased stock stored at −20°C in 1 mL DMSO. Maximum time for storage 6 months.
Tris-HCl 1 M pH 6.8 and 7.5	Dissolve 121.1 g Tris in 800 mL ddH_2_O, adjust to pH 6.8 or 7.5 with concentrated HCl, filter with 0.22 μm filter. Maximum time for storage 1 month.
Triton X-100 10% (v/v)	Add 4 mL Triton X-100 to 36 mL ddH_2_O, filter with 0.22 μm filter, and store at 4°C. Maximum time for storage 6 months.
Uranyl acetate 2% solution	Dissolve 0.8 g of uranyl acetate powder in 40 mL ddH_2_O by stirring on a magnetic stirrer for 24 h. Filter solution through a 0.22 μm filter with a syringe. Aliquot into Eppendorf tubes and place them in a rack protected from light with aluminum foil. Store at 20°C–25°C or at 4°C. Appropriate safety guidelines must be followed when working with radioactive material. Maximum time for storage 1 year.
β-glycerophosphate 1 M pH 7.6	Dissolve 21.6 g β-glycerophosphate in 100 mL ddH_2_O. Adjust to pH 7.6 with KOH before topping up the volume. Filter with 0.22 μm filter and store at 4°C. Maximum time for storage 6 months.

**Table:** Acetate Nuclear Isolation Buffer (ANIB 2x)

Components	Stock concentration	Amount	Final concentration
HEPES-KOH pH 7.6	1 M	10 mL	100 mM
KOAc	4 M	5 mL	200 mM
Mg(OAc)_2_	1 M	2 mL	20 mM
Mg-ATP	250 mM	2 mL	5 mM
β-glycerophosphate	1 M	5 mL	50 mM
Spermidine	500 mM	200 μL	1 mM
Spermine	500 mM	60 μL	0.3 mM
APR	10 mg/mL	20 μL	2 μg/mL
PEP	10 mg/mL	20 μL	2 μg/mL
LEU	10 mg/mL	20 μL	2 μg/mL
Top up to 100 mL with ddH_2_O			

ANIB 2x is immediately filtered with a 0.22 μm filter. It is typically prepared in advance of chromatin isolation and stored at −20°C. Maximum time for storage 1 year.

**Table:** Acetate Nuclear Isolation Buffer (ANIB)

Components	Stock concentration	Amount	Final concentration
ANIB 2x	2x	5 mL	1x
Triton X-100	10%	100 μL	0.1%
2-Chloracetamide	500 mM	100 μL	5 mM
PMSF	100 mM	10 μL	0.1 mM
Top up to 10 mL with ddH_2_O			

ANIB is prepared fresh and used the same day. Remember that 2-Chloracetamide is prepared fresh while making ANIB. Keep it on ice all the time during the experiment.

**Table:** Acetate Nuclear Isolation Buffer with sucrose (ANIB-sucrose)

Components	Stock concentration	Amount	Final concentration
ANIB 2x	2x	1 mL	1x
Triton X-100	10%	20 μL	0.1%
2-Chloracetamide	500 mM	20 μL	5 mM
PMSF	100 mM	2 μL	0.1 mM
Top up to 2 mL with sucrose 40%			

ANIB-sucrose is prepared fresh and used the same day. Remember that 2-Chloracetamide is prepared fresh while making ANIB. Keep it on ice all the time during the experiment.

**Table:** Cysteine solution (CS)

Components	Stock concentration	Amount	Final concentration
L-cysteine	N/A	44 g	200 mM
EGTA	N/A	3.8 g	5 mM
Adjust to pH 7.6 with KOH and top up to 2 L with ddH_2_O			

Cysteine solution is prepared fresh and used the same day at 20°C–25°C.

**Table:** Extraction Buffer (EB)

Components	Stock concentration	Amount	Final concentration
HEPES-KOH pH 7.6	1 M	5 mL	50 mM
KCl	3 M	1.66 mL	50 mM
MgCl_2_	1 M	0.5 mL	5 mM
β-mercaptoethanol	14.6 M	13.7 μL	2 mM
Top up to 100 mL with ddH_2_O			

EB is prepared the day before needed and stored at 4°C.

**Table:** Extraction Buffer-glycerol (EB-glycerol)

Components	Stock concentration	Amount	Final concentration
Glycerol	≥99.5%	30 mL	30% (v/v)
HEPES-KOH pH 7.6	1 M	5 mL	50 mM
KCl	3 M	1.66 mL	50 mM
MgCl_2_	1 M	0.5 mL	5 mM
β-mercaptoethanol	14.3 M	13.7 mL	2 mM
Top up to 100 mL with ddH_2_O			

EB-glycerol is prepared the day before needed and stored at 4°C.

**Table:** Energy Regenerator (ER)

Components	Stock concentration	Amount	Final concentration
Creatine phosphate	N/A	3.06 mg	306 mg/mL
Creatine phosphokinase	N/A	36 mg	3.6 mg/mL
HEPES-KOH pH 7.6	50 mM	10 mL	50 mM

ER is immediately filtered with a 0.22 μm filter. It is typically prepared on a different day than the egg extract protocol initiation and stored at −20°C in 50 μL aliquots. Maximum time for storage 1 year.

**Table:** Immunoprecipitation Elution Buffer (IP-EB)

Components	Stock concentration	Amount	Final concentration
ATP	100 mM	50 μL	1 mM
HEPES-KOH pH 7.6	1 M	4.8 mL	25 mM
IGPAL CA-630	≥99.5%	1 μL	0.02% (v/v)
KOAc	4 M	125 μL	100 mM
Mg(OAc)_2_	1 M	25 μL	5 mM

IP-EB is immediately filtered with a 0.22 μm filter. It is prepared fresh and used on the day. Keep it on ice all the time during the experiment.

**Table:** Licensing Factor Buffer (LFB1/50)

Components	Stock concentration	Amount	Final concentration
DTT	1 M	2 mL	2 mM
EGTA	0.5 M	2 mL	1 mM
HEPES-KOH pH 8	1 M	40 mL	40 mM
KCl	3 M	16.6 mL	50 mM
KH_2_PO_4_ pH 8.0	0.5 M	40 mL	20 mM
MgCl_2_	1 M	2 mL	2 mM
Sucrose	N/A	100 g	10% (w/v)
Top up to 1 L with ddH_2_O			

LFB 1/50 is immediately filtered with a 0.22 μm filter. It can be stored at 4°C for short periods of time or frozen at −20°C. Maximum time for storage 1 year.

**Table:** Mg-ATP

Components	Stock concentration	Amount	Final concentration
ATP	N/A	2.75 g	250 mM
MgCl_2_	N/A	1.02 g	250 mM
Adjust to pH 8.0 with NaOH and top up to 20 mL of ddH_2_O			

Mg-ATP is stored at −20°C. Maximum time for storage 1 year.

**Table:** Marc’s Modified Ringer’s (MMR 10x)^11^

Components	Stock concentration	Amount	Final concentration
CaCl_2_	1 M	60 mL	20 mM
EDTA	0.5 M	6 mL	1 mM
HEPES	N/A	35.78 g	50 mM
KCl	N/A	4.5 g	20 mM
MgCl_2_	1 M	30 mL	10 mM
NaCl	N/A	175.35 g	1 M
Adjust to pH 7.8 with NaOH and top up to 3 L with ddH_2_O			

MMR 10x is prepared fresh and used the same day. It is also used to prepare MMR 1x which is stored at 4°C and used cold the day after.

**Table:** MS-222

Components	Stock concentration	Amount	Final concentration
MS-222	N/A	2 g	0.2% (w/v)
NaHCO_3_	N/A	5 g	0.5% (w/v)
Adjust to pH 7.5 with NaOH and top up to 1 L with ddH_2_O			

MS-222 is prepared fresh and used the same day.

**Table:** Proteinase K

Components	Stock concentration	Amount	Final concentration
ddH_2_O	N/A	480 μL	N/A
Glycerol	≥99.5%	500 μL	50% (v/v)
Proteinase K	N/A	20 mg	20 mg/mL
Tris-HCl pH 6.8	1 M	20 μL	20 mM

Proteinase K is stored at −20°C. Maximum time for storage 1 year.

**Table:** Stop buffer C 10x

Components	Stock concentration	Amount	Final concentration
EDTA	0.5 M	10 mL	5 mM
SDS	N/A	0.5 g	0.5% (w/v)
Tris-HCl pH 7.5	1 M	20 mL	20 mM
Top up to 100 mL with ddH_2_O			

Stop buffer C 10x is stored at 37°C. Maximum time for storage 6 months.

**Table:** SuNaSp

Components	Stock concentration	Amount	Final concentration
HEPES	N/A	0.36 g	15 mM
NaCl	N/A	0.44 g	75 mM
Spermidine	0.5 M	100 μL	0.5 mM
Spermine	0.5 M	30 μL	0.15 mM
Sucrose	N/A	8.6 g	250 mM
Adjust to pH 7.6 with KOH and top up to 100 mL with ddH_2_O			

SuNaSp is prepared fresh and used on the day. Keep it on ice all the time during the experiment.

**Table:** SuNaSp-BSA

Components	Stock concentration	Amount	Final concentration
BSA	30% (w/v)	10 mL	3%
HEPES	N/A	0.36 g	15 mM
NaCl	N/A	0.44 g	75 mM
Spermidine	0.5 M	100 μL	0.5 mM
Spermine	0.5 M	30 μL	0.15 mM
Sucrose	N/A	8.6 g	250 mM
Adjust to pH 7.6 with KOH and top up to 100 mL with ddH_2_O			

SuNaSp-BSA is prepared fresh and used on the day. Keep it on ice all the time during the experiment.

**Table:** Un-activating Extraction Buffer (UEB)

Components	Stock concentration	Amount	Final concentration
DTT	N/A	308.5 mg	1 mM
EGTA	0.5 M	20 mL	5 mM
HEPES	N/A	23.8 g	50 mM
KCl	N/A	7.46 g	50 mM
MgCl_2_	1 M	10 mL	5 mM
Adjust to pH 7.6 with KOH and top up to 2 L with ddH_2_O			

UEB is prepared fresh the day before use and stored at 4°C. Add DTT in the buffer just before use it (Point 8.f).

## Step-by-step method details

### *Xenopus laevis* demembranated sperm nuclei preparation

**Timing: Day 1: 2 h (for step 1)**

**Timing: Day 2: 8 h (for step 2)**

1. Folligon injection. Day 1.
  a. Prime male frogs with 100 IU of Folligon (PMSG) 5–9 days before testes are removed to increase yield of sperm.

**Figure 4:**
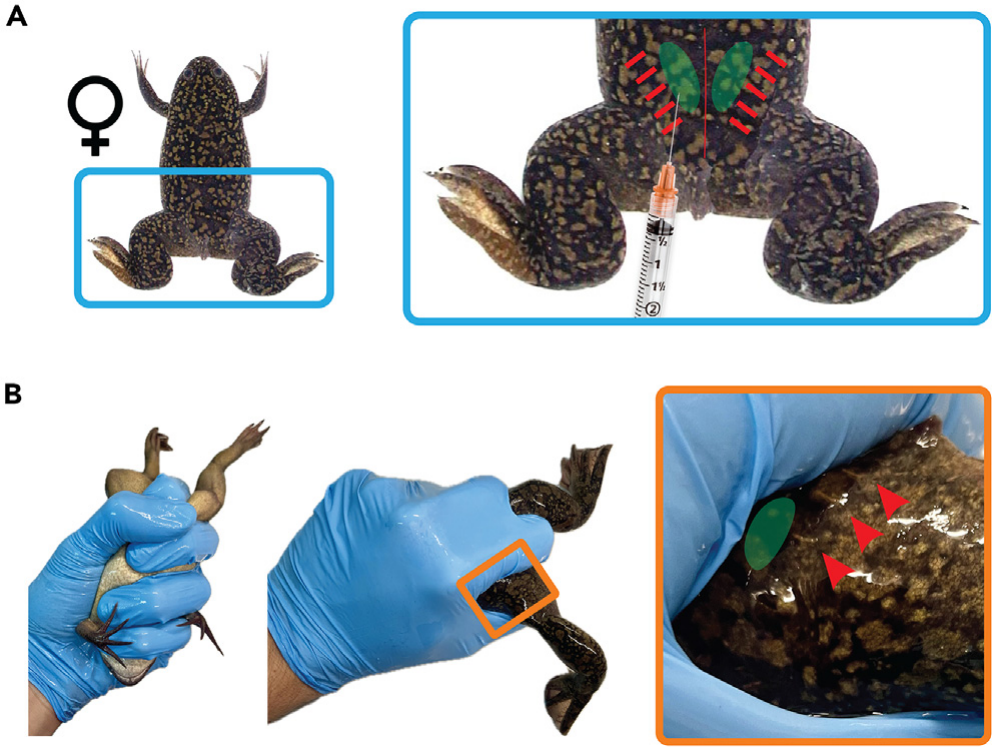
Handling and injection of *Xenopus laevis* Both females and males are injected into the dorsal lymph sac. (A) The area for injection corresponding to the dorsal lymph sac is highlighted in green. Red indicates the lateral line system, resembling stitches on the frog skin. These are sensorial organs, therefore, avoid injecting directly around them. (B) Pictures of how to handle a frog for injection and how to expose the area to inject.

***Note:*** Usually, 15 male frogs are used per procedure. As in the case of females, male frogs are injected into the dorsal lymph sac using a 1 mL syringe with a 25-G needle (Figure 4).

2. Euthanasia of frog males. Frogs are culled using deep anesthesia and cessation of blood circulation. Day 2.
  a. Place frogs in groups of 3 into individual chambers containing 0.5 L MS-222 anesthetic solution and process them in parallel.
    ***Note:*** When frog’s heads drop under water and they stop moving when touched, check for reflexes by placing a forefinger deep into their mouth. If the frog gives a reflex choking response, it is still conscious, therefore keep it in MS-222 and check every min or so until the reflex is lost.
    ***Note:*** It is practical for 2–3 people to process frogs in parallel to speed up the procedure.
  b. Remove the frogs from the MS-222 and place them on their back on the bench.
    ***Note:*** Testes are localized in the lower abdominal region. They are most easily accessible when the frog is placed on its back and entered ventrally (Figure 5A).

**Figure 5:**
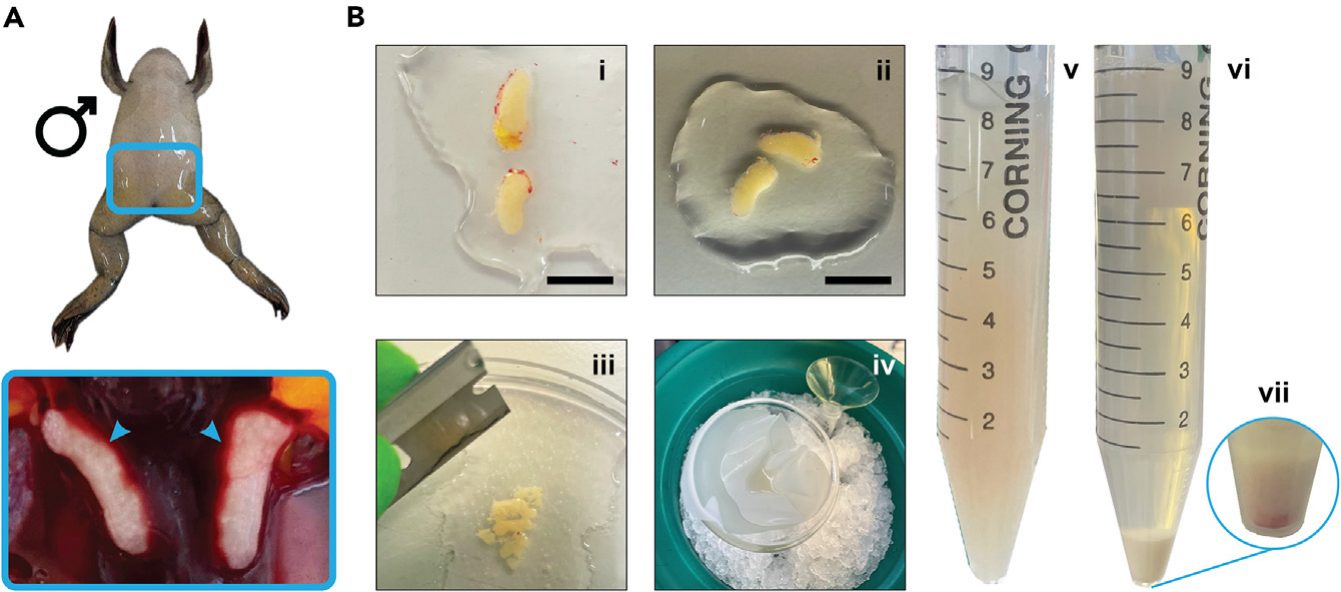
Images of demembranated sperm nuclei preparation (A) *Xenopus laevis* male with highlighted the area where testes are located. Insert: Picture of testes surrounded by other tissues. (B) Dissected testis with blood vessels (i). Cleaned testis (ii), ready to be chopped (iii) and filtered (iv). The filtered material (v) is subsequently centrifuged (vi) and eventual erythrocytes (vii) removed before collection and storage. Scale bar 1 cm.
  c. Cut open the skin, locate the heart in the top right part of the torso and cut the arteries leading to the heart, leading to cessation of circulation and death.
3. Testes dissection.
  a. Locate the testes as quickly as possible.
    ***Note:*** They are bean/egg shaped and ivory in color, and typically of 0.75–1.5 cm in length (Figure 5Bi-ii).
  b. Dissect the testes as quickly as possible, carefully avoiding damaging them and place in a falcon tube containing cold EB on ice.
    ***Note:*** Although most frogs have two testes, it is possible that some of them have one, or more rarely, three testes. Moreover, testes may have different sizes within the same animal.
4. Demembranated sperm nuclei preparation.
  a. Wash testes with ∼2 mL EB solution in a Petri dish.
  b. Clean them by carefully removing any blood vessels and extraneous tissue using dissection forceps (Figure 5 Bi-ii)
    **CRITICAL:** Be careful not to burst the testes.
  c. Transfer two cleaned testes to a new Petri dish containing 1 mL EB solution.
  d. Chop them as finely as possible with a razor blade until no particles are visible (Figure 5Biii). (Repeat with all testes).
    ***Note:*** Usage of homogenizers leads to poorer sperm release from the tissue. Manual chopping using a razor blade gives superior yield.
  e. Chopped tissue is filtered through a 25 μm mesh nylon filter material mounted on a small funnel placed in a 50 mL Falcon tube (Figure 5Biv). Keep it all on ice.
    ***Note:*** The filtered material should look quite cloudy.
  f. Transfer the filtered material to a 15 mL Falcon tube.
  g. Centrifuge at 2,000 × *g* for 5 min at 4°C (Megafuge 16 with TX-400 swinging bucket rotor).
    ***Note:*** If the supernatant appears cloudy after centrifugation, centrifuge the supernatant once more. Combine the resulting pellet fractions (Figure 5Bv-vi). Troubleshooting 1.
  h. Resuspend pelleted sperm nuclei in 0.5 mL of SuNaSp per testis at 20°C–25°C.
  i. Supplement with 25 μL of lysolecithin solution per testis.
  j. Incubate for 5 min at 20°C–25°C.
  k. Determine the demembranation of the sperm.
    i. Mix 1 μL of the sperm sample with 1 μL of SYTOX Orange on a glass slide.
    ii. Imaging the sample with UV microscopy (e.g., EVOS XL).
      ***Note:*** Demembranated sperm stained with SYTOX Orange should look like elongated S-shaped filaments of ∼20 μm in length and ∼2 μm in diameter, stained bright red. Contrarily, non-demembranated sperm should not be stained. If less than 95% of the sperm is demembranated, spin the mixture again, resuspend the pellet with fresh SuNaSp and repeat lysolecithin treatment (Point 4.i).
  l. Once demembranated, centrifuge the sperm homogenate at 2,000 × *g* for 5 min at 4°C (Megafuge 16 with TX-400 swinging bucket rotor).
  m. Quench lysolecithin by resuspending the pellet in 500 μL per testis of SuNaSp-BSA.
  n. Wash demembranated sperm nuclei twice with EB solution.
  o. Resuspend the pellet in 100 μL EB-glycerol per testis.
5. Demembranated sperm nuclei quantification.
  a. To count the sperm, make a small aliquot of the resuspended sperm (Point 4o) diluted 1:100 in EB.
  b. Use a haemocytometer to count the number of sperm and large somatic-type nuclei.
  c. Dilute the stock in EB-glycerol to a final concentration of 1000 ng DNA/μL.
  d. Prepare aliquots of 100 μL and store at −80°C.

**CRITICAL:** Thoroughly mix the sperm prep before and during aliquoting to avoid sedimentation of sperm DNA and variable concentration across aliquots.

**Pause point:** Once prepared the demembranated sperm aliquots can be stored for years and tested further at any time.

### *Xenopus laevis* Low-Speed Supernatant Egg Extract preparation

**Timing: Day 1: 1.5 h (for step 6)**

**Timing: Day 2: 2 h (for step 7)**

**Timing: Day 3: 4 h (for step 8–9)**

6. Folligon injection. Day 1.
  a. Prime 10 female frogs with 100 IU of Folligon 2–7 days before eggs are required.

***Note:*** Priming is performed to increase the number of stage 6 (mature) oocytes. The injection is performed as shown in Figure 4. Frogs are not fed for up to 5 days before Chorulon injection to avoid feces and food contamination amongst the eggs.

7. Chorulon injection. Day 2.
  a. The day before eggs are required, inject frogs with 500 IU of Chorulon.
  b. Placed frogs in laying tanks containing 2.5 L MMR 1x at 19°C–23°C.

***Note:*** Usually, frogs are injected at 16:00 and start laying eggs overnight. MMR inhibits premature activation of the eggs once laid. The injection is performed as shown in Figure 4. A temperature over 23°C leads to a high proportion of apoptotic eggs.

**Figure 6:**
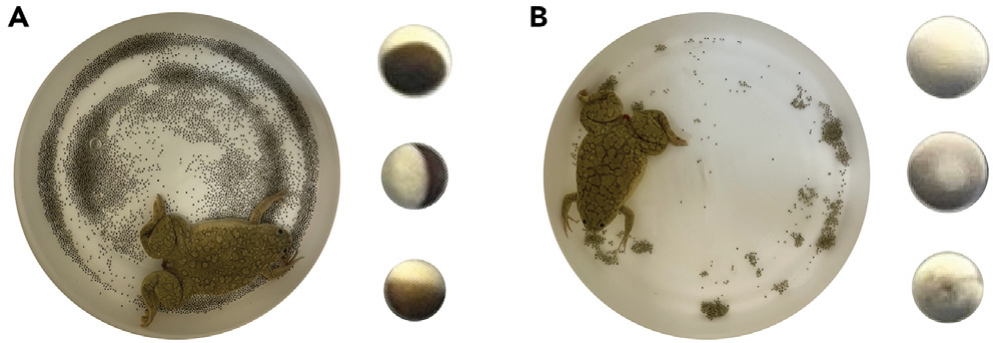
Images of eggs collection (A) Good quality eggs are inactivated and arrested in meiosis II metaphase. They are individual, with clear dark animal pole and light vegetal pole, and surrounded by clear buffer. (B) Bad quality eggs may be characterized by discolored or speckled yolks, white bloated eggs, stringy eggs, small size, dirty jelly coats with debris adhering to the surface.
  b. Rinse the eggs with 1 L MMR 1x and remove as much buffer as possible after rinsing.
  c. Dejelly the eggs by adding cysteine solution (Figures 7A and 7B).
    **CRITICAL:** When eggs start dejellying, the buffer becomes cloudy. Leave cysteine acting for a few min while gently swirling the eggs before exchange with fresh cysteine solution. Use cysteine solution until it remains clear, and the eggs are compacted. Do not leave the eggs in cysteine solution for more than 10 min.

8. Egg collection. Day 3.
  a. Collect eggs after 16–18 h post injection into a 1 L glass beaker.
    ***Note:*** Only good quality eggs are collected (Figure 6). Eggs from tanks containing too many activated or apoptotic eggs (white, swollen, stringy or floating eggs) are discarded (Figure 6)
    **CRITICAL:** Do not use plastic beakers.

**Figure 7:**
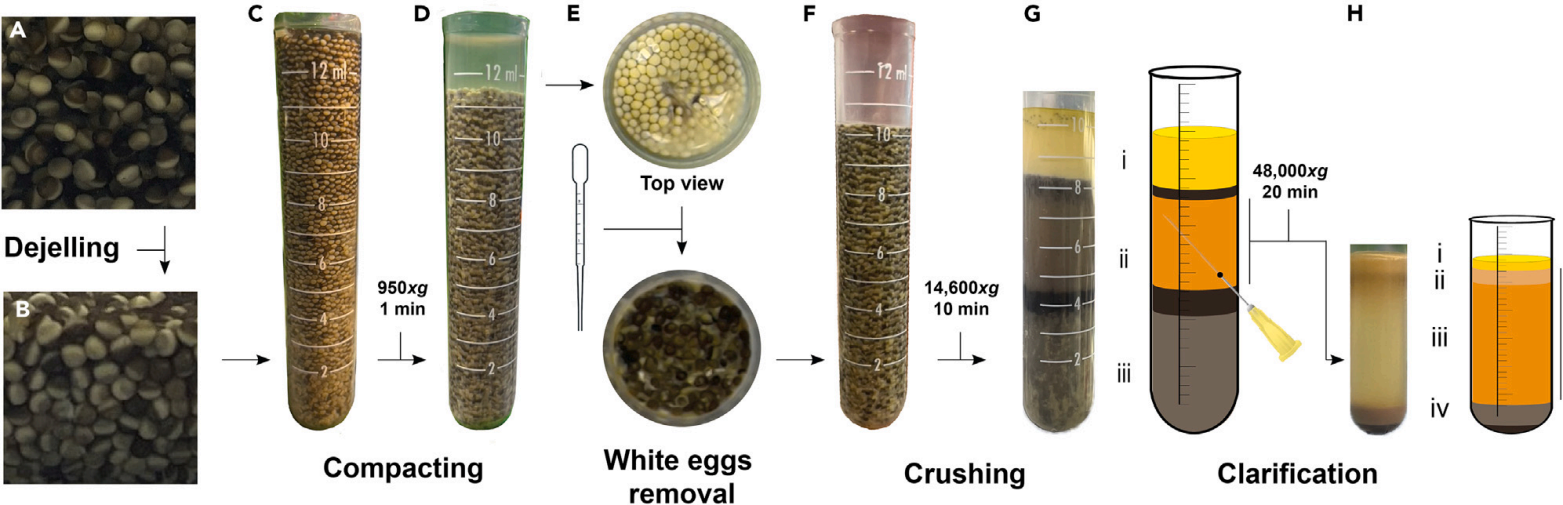
Images of the major steps of LSS preparation (A) Rinsed eggs with jelly layer still present. (B) Eggs after dejellying are compacted at the bottom of the beaker. (C) Dejellied eggs transferred into a tube. (D) Compacted eggs with presence of buffer and apoptotic eggs on the top of the tube. (E) Floating apoptotic eggs (white) are removed using a plastic Pasteur pipette. (F) Compacted eggs ready to be spin-crushed. (G) Egg extract, after crushing spin. (i) lipid layer, (ii) crude cytoplasm, (iii) yolk platelets. (H) Egg extract, after clarifying spin. (i) lipid layer, (ii) membranous layer, (iii) cytoplasm, (iv) mitochondria, residual yolk platelets and insoluble material.
  d. Rinse with 1 L MMR 1x taking out any white or swollen eggs with a plastic Pasteur pipette.
  e. Pour off as much MMR as possible.
  f. Start adding UEB buffer (with added DTT) and continue to remove all the white eggs using a plastic Pasteur pipette.
    ***Note:*** Apoptotic white eggs are larger and less dense and collect on the top of egg mass. Swirling and vortex creation brings all white eggs together for easy removal.
9. Egg extract preparation.
  a. Pour the eggs into 14 mL round bottom tubes containing 1 mL UEB buffer supplemented with APR, LEU and PEP to 10 μg/mL (1000x), as well as cytochalasin D to 50 μg/mL (200x). Fill the tubes to the top (Figure 7C).
  b. Packed the eggs by centrifuging at 950 × *g* for 1 min at 20°C (Avanti J-E with JS-13.1 swinging bucket rotor) (Figure 7D).
  c. Remove any buffer from the top of packed eggs using a plastic Pasteur pipette (Figures 7E and 7F).
    **CRITICAL:** After packing, white eggs tend to float and create a white layer on top of the eggs. This must be gently removed using a plastic Pasteur pipette before the next step (Figures 7E and 7F). These white eggs crush very easily, be very careful not to leave remnants of the liquid behind.
  d. Spin-crush the eggs by centrifuging at 14,600 × *g* for 10 min at 20°C (Avanti J-E with JS-13.1).
    ***Note:*** This separates the eggs into a gray insoluble pellet, a brownish cytoplasm, and a yellow lipid plug (Figure 7G).
  e. Piercing the tube using a 20-G needle.
  f. Carefully collect the cytoplasmic layer using a new 20-G needle and 1 mL syringes as shown in Figure 7G.
  g. Collect the extract into a 50 mL Falcon tube placed on ice.
    ***Note:*** Take as much extract as possible (Figure 7Gii), without worrying too much about collecting the layers above, but try to avoid the layers below.
  h. Supplement the extract with protease inhibitors APR, LEU and PEP to 10 μg/mL (1000x), CytD to 10 μg/mL (1000x) and 15% v:v LFB 1/50.
  i. Gently but thoroughly mix with a plastic Pasteur pipette.
  j. Transfer the extract to 5 mL ultracentrifuge tubes and centrifuge at 48,000 × *g* for 20 min at 4°C (Optima LE-80K with SW50.1) (Figure 7H).
  k. Remove the lipid plug from the top of the tube with a clean spatula (Figure 7Hi).
  l. Collect all the golden cytoplasmic layers (Figure 7Hiii), include the membrane layer which is just below the lipid plug (Figure 7Hii), but avoid the gray mitochondrial layer just below the golden cytoplasmic layer (Figure 7Hiv).
    **CRITICAL:** While taking the cytoplasm use a source of light and ensure that the darker layer just below does not get perturbed during the process. That layer contains mitochondria, which will promote apoptosis in the extract upon freezing/thawing (Figure 7Hiii).
  m. Add 50% sterile glycerol to a final concentration of 1% v:v and mix gently.
  n. Arrange 4 plastic Petri dishes in a polystyrene container and fill them with liquid nitrogen.
  o. Using a pipette with a pre-cut tip, drop the extract in the liquid nitrogen producing frozen extract beads.
  p. Transfer the beads into an appropriate number of cryo-tubes and store at −80°C for years.
    **Pause point:** The new batch of LSS can be tested at any point after freezing with liquid nitrogen. In general, we test frozen LSS to make sure that stored extract is capable of synthesizing DNA, and the freezing process did not kill its activity (through bursting of the retained mitochondria upon thawing).

### Large scale chromatin isolation and FLAG immunoprecipitation

**Timing: 20 min (for step 10)**

**Timing: 1 min (for step 11)**

**Timing: 70 min (for step 12)**

**Timing: 40 min (for step 13)**

**Timing: 4 h (for step 14)**

10. Egg extract activation.
  a. Transfer 4 mL of frozen extract beads to a tube to thaw.
  b. Add ER (40x), CHX to 0.25 mg/mL (40x) and CaCl_2_ to 0.3 mM (167x).
  c. Add Cdc45-FLAG_5_ to 1 μM and mix thoroughly.
    **CRITICAL:** To avoid diluting the extract too much, it is crucial to use a highly concentrated solution of recombinant Cdc45-FLAG_5_. Addition of over 1/10 volume of solution into LSS egg extract can result in its inability to form nuclei and/or carry out DNA replication. In our study we used a stock of recombinant Cdc45-FLAG_5_ at 47 μM.
    ***Note:*** We are using Cdc45-FLAG_5_ to isolate CMG complexes, but other tagged recombinant proteins can be used if other complexes are to be isolated.
  d. Leave the extract activating on the bench at 20°C–25°C for 15 min.
11. Replication reaction conditions.
  a. Add aphidicolin and caffeine to the extract with a final concentration of 40 μM (200x) and 5 mM (20x), respectively.
  b. Add DNA to the extracts to a final concentration of 20 ng/μL and mix thoroughly.
  c. Start the timer.
  d. Incubate at 23°C.
12. Chromatin isolation.
  a. Isolate chromatin after 60 min.
  b. To do so, split the extract in four aliquots of 1 mL using 15 mL Falcon tubes.
  c. Add 5 mL of ANIB to each aliquot and mix thoroughly.
  d. Underlay with 1 mL ANIB-sucrose.
    **CRITICAL:** Place the pipette tip to the bottom of tube and gently deposit ANIB-sucrose buffer beneath ANIB. Do not mix the two buffers allowing the formation of a sucrose cushion.
  e. Spin at 2,500×*g* for 5 min at 4°C (Megafuge 16 with TX-400 swinging bucket rotor). Troubleshooting 2.
  f. Remove the whole ANIB buffer phase with a pipette without perturbing the sucrose phase.
  g. Wash the sides of the tube with 1 mL ANIB without perturbing the ANIB-sucrose phase.
  h. Remove the ANIB used for washing, as well as part of the sucrose cushion interface to reduce contaminations. (Repeat 2–3 times).
  i. Remove as much as possible of the ANIB-sucrose without disrupting the chromatin pellet.
  j. Transfer the pellet into a microfuge tube using a pipette.
  k. Resuspend chromatin with 600 μL of fresh ANIB-sucrose via pipetting.
    ***Note:*** If it is difficult to resuspend the chromatin pellet, using a pre-cut tip, keep pipetting vigorously avoiding producing bubbles until the largest pieces are broken down. It is fine if there are some visible floating bits of chromatin.
13. Chromatin sonication and digestion.
  a. Sonicate the sample for 5 min (15 s ON – 15 s OFF) at low setting in a Bioruptor sonicator.
  b. Add benzonase to 0.4 U/μL and incubate on a roller at 20°C–25°C for 30 min.

***Note:*** During this time go to step 14.a and prepare accordingly.

***Note:*** At this point, the sample should look transparent with no visible chromatin particles. Troubleshooting 3.

14. FLAG immunoprecipitation.
  a. While the chromatin is digesting with benzonase (step 13.b) prepare your FLAG beads.
    i. Transfer 100 μL FLAG beads to a microtube.
    ii. Using a magnetic rack, wash beads with 1 mL PBS, PBST and PBS.
    iii. Incubate beads in 990 μL PBS and 10 μL BSA 30% for 10 min at 20°C–25°C on a rotary incubator.
    iv. Using a magnetic rack, wash beads with 1 mL ANIB and ANIB-sucrose.
    v. Leave beads in fresh ANIB-sucrose until needed for IP.
  b. Take 5 μL of sample from step 13.b to make Chromatin sample (Ch).
  c. Spin the rest of the digested chromatin at 16,900 × *g* for 2 min at 4°C (Eppendorf 5418R). Troubleshooting 4.
  d. Transfer the supernatant into a fresh microtube and take 5 μL out, which constitute the Input sample (Inp).
  e. Resuspend pellets from the previous step in 600 μL of ANIB, and use 5 μL of the suspension to make the Chromatin Pellet sample (ChPel).
  f. Incubate ∼600 μL supernatant from step 14.c with 100 μL of previously prepared FLAG beads (point 14.a) on rotary incubator for 1.5 h at 4°C.
    ***Note:*** This step may need to be optimized for complexes other than CMG.
  g. Using the magnetic rack, separate FLAG beads from the solution and transfer the latter to a fresh microtube.
  h. Take out 5 μL of sample at step 14.g to make Flowthrough/Depleted sample (DP).
  i. Wash the beads five times via resuspending and gently mix via inverting the tube a few times with:
    i. 1 mL ANIB-sucrose.
    ii. 1 mL ANIB-sucrose with 0.1% Triton X-100.
    iii. 1 mL ANIB-sucrose.
    iv. 1 mL IP-EB. (Repeat 2 times).
  j. Remove all washing buffer.
  k. Elute the complex by adding 200 μL IP-EB and 250 μM triple FLAG peptide.
  l. Allow elution for 2 h at 4°C on a rotary mixer.
    ***Note:*** This step can be further optimized reducing the incubation time.
  m. Using the magnetic rack, separate FLAG beads from the solution and transfer the latter to a fresh microtube.
  n. Take 10 μL of sample to make the immunoprecipitated sample (IP).
    ***Note:*** Sample at step 14.m will be deposited on EM grids for negative staining, and ultimately cryo-EM.
    ***Optional:*** Incubate the beads with 50 μL of IP-EB and 1 μL triple FLAG peptide for 5 min at 4°C. Then prepare a second elution sample (IP2) by taking 10 μL of it.
  o. Add 25 μL LB 1x to samples Ch, ChPel and DP, while add 10 μL LB 2x to IP.
  p. Incubate samples at 95°C for 5 min.
  q. Run SDS-PAGE and silver staining (Point 15).

### Analysis of purified replisomes

**Timing: 2.5 h (for step 15)**

**Timing: 30 min to 1 h (time will depend on the number of grids) (for step 16)**

**Timing: 20 min to 1 h (time will depend on the number of grids) (for step 17)**

**Timing: 1–1.5 h (time will depend on the number of grids) (for step 18)**

15. SDS-PAGE and silver staining.
  a. Load 20 μL of the samples generated in step 14 (Ch, ChPel, Inp, DP and IP) on a NuPAGE 4%–12% Bis-Tris gradient 15 well gel and run at 200 V for 45 min (Bio-Rad PowerPac).
  b. Stain the gel using SilverQuest kit, or any other suitable silver staining kit.

***Note:*** The gel is fixed, sensitized, stained, and developed with provided solutions. More details are available from the manufacturer (link). An equivalent silver staining procedure can be used.

**CRITICAL:** To reduce the chance of sample loss due to protein complexes falling apart over time, we suggest skipping point 15 and proceeding directly with negative staining (Point 16).

16. Sample preparation for negative stain electron microscopy (NS-EM).
  a. Glow discharge grids coated with continuous carbon (EM Resolutions C300Cu) with a glow discharger or plasma cleaner. We used a GloQube Plus unit (Quorum Technology), with the following parameters: 25 mA current, 60 s duration and negative polarity.
    ***Note:*** Make sure that grids are placed in the glow discharge unit with their carbon film surface facing up. Glow discharging will make this surface hydrophilic, enabling uniform spreading of the aqueous sample and particle absorption on carbon.
  b. Cool down 2% uranyl acetate (UA) solution aliquot by placing it on ice for 5–10 min.
  c. Spin UA at 20,000 × *g* for 4 min at 4°C on a benchtop centrifuge (Eppendorf 5424R) to separate any precipitates and keep the UA solution at 20°C–25°C during sample preparation.
    ***Note:*** Protect UA from light to avoid precipitation. Keep UA solution aliquots in Eppendorf tubes wrapped in aluminum foil. While performing steps that involve UA manipulation, follow safety guidelines for working with radioactive material, including safe disposal of any contaminated item.
  d. Pipet three 30 μL droplets of UA solution onto a piece of parafilm from the top of the Eppendorf tube. Take care not to perturb any precipitate that might be present at the bottom of the tube (Figure 8A).

**Figure 8:**
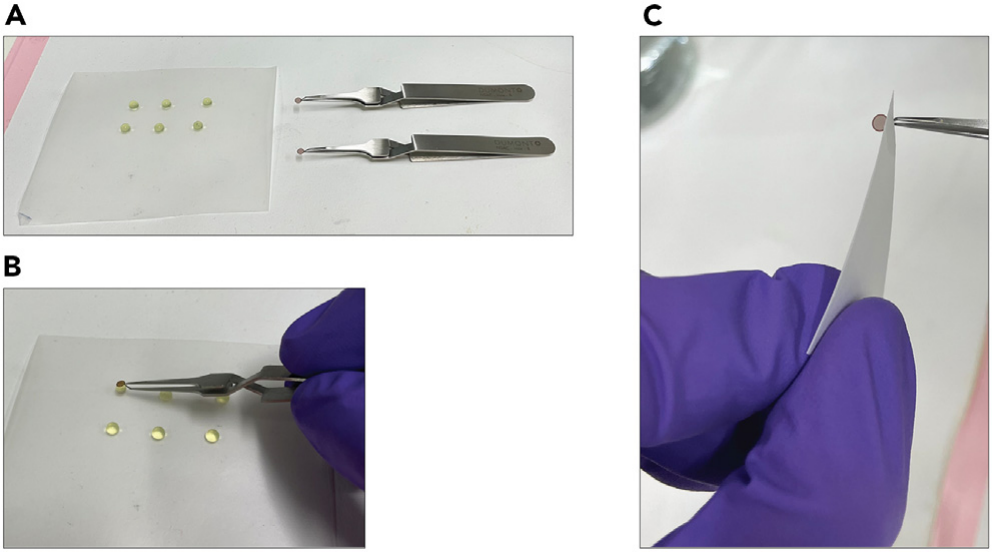
Negative staining of the grids (A) Set-up for negative staining with grids in tweezers and UA droplets on parafilm. (B) Staining of a grid on the first UA droplet. (C) Blotting away residual UA solution.
  e. Hold a grid by the rim using crossover locking tweezers. Place the tweezers on the bench with the glow discharged carbon surface of the grid facing upwards (Figure 8A).
  f. Apply 4 μL of the sample (from point 14.L) on the top surface of the grid and wait 1.5 min.
    ***Note:*** Incubation time can vary from one to two min. Increased incubation time can lead to some increase of particle absorption on carbon.
  g. Blot away the sample by touching the edge of the grid using Whatman filter paper (as shown in Figure 8C).
  h. Using the locked tweezers invert the grid and put it in contact with the first UA droplet, gently swirling it for 20 s, without ever breaking surface tension. Make sure that only one side of the grid is exposed to the stain solution in the process (Figure 8B).
  i. Quickly switch to the second UA droplet, swirling the grid for another 20 s and then repeat the same operation on the third droplet. No blotting should occur while transitioning from droplet to droplet.
  j. After exposure to the third UA droplet, gently blot away all residual UA solution from the grid by touching the rim of the grid with Whatman filter paper (Figure 8C) and leave the grid locked with tweezers to dry on the bench.
  k. Once the grid is dry (2–3 min wait), place it into a grid box for room temperature storage.
  l. Safely dispose all contaminated material.
  m. If particle quality evaluated by NS-EM is satisfactory, proceed with plunge freezing on the same day. Troubleshooting 5.
17. Graphene oxide (GO) application onto the grids for cryo-electron microscopy (cryo-EM).
  a. Add 10 μL of the graphene oxide stock suspension (2 mg/mL) to 80 μL of Milli-Q water and resuspend the mixture by pipetting up and down repeatedly.
  b. Spin down remaining larger GO agglomerates using a benchtop centrifuge (Eppendorf 5424R) at 650 × *g* for 1.5 min, at 20°C–25°C.
  c. Carefully transfer the clear top layer of the GO suspension into a new Eppendorf tube without touching the bottom of the tube. This clear GO suspension will be used for application on the grid.
  d. Glow discharge grids (UltrAuFoil R1.2/1.3, Au 300 mesh) using a glow discharger or plasma cleaner. We use a GloQube Plus unit with the following parameters: 45 mA current, 5 min duration and negative polarity.
    ***Note:*** Make sure that the grids are placed top side up during glow discharging. The manufacturers pack grids with their top sides, where the sample should be applied, facing the center of the grid box.
  e. Hold a grid by the rim with crossover locking tweezers and place the tweezers on the bench with the glow discharged top grid surface facing upwards.
  f. Pipet 3 separate droplets of 20 μL Milli-Q water per grid on a clean piece of parafilm.
  g. Pipet 4 μL of the clear GO suspension on the top side of the grid and wait for 2–3 min.
  h. Turn the grid held with the tweezers upside down and blot away GO by touching a clean Whatman filter paper with the edge of the grid.
  i. Immediately pick up one water droplet from the parafilm with the GO-exposed surface of the grid. The grid should be held upside down using the locked tweezers throughout this process. Without delay, blot the water droplet by touching a dry (unused) area of the Whatman filter paper.
  j. Repeat the previous step, using the second water droplet.
  k. Turn the grid back to the original orientation, with top side facing upwards, pick up the last water droplet with the bottom side of the grid (the side that was not exposed to GO) and blot it away using the Whatman filter paper.
    Let the grid dry for 5 min on the tweezers. Store it on a piece of clean Whatman filter paper, protected inside a Petri dish until ready for freezing.
    ***Note:*** GO application should be performed within a few hours before sample application and plunge freezing.
18. Sample application and plunge freezing grids using a Vitrobot.
  a. Attach humidifier to the Vitrobot (FEI Thermo Scientific, Vitrobot Mark IV) and inject 60 mL water with a syringe (Figure 9A). After filling the cylinder, remove any air by pulling back on the syringe plunger.

**Figure 9:**
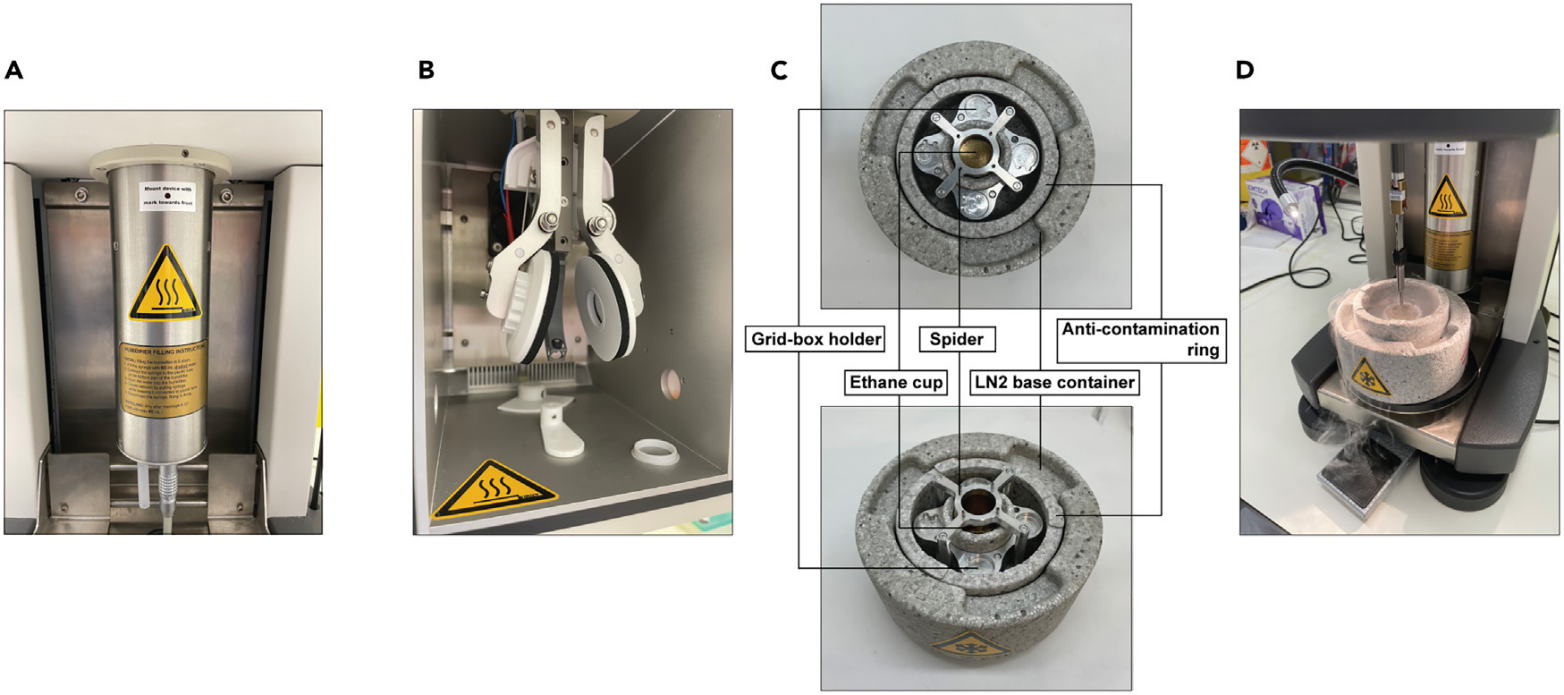
Set-up for plunge freezing (A) Humidifier attached to the Vitrobot. (B) Circular blotting paper attached to blotting pads inside the Vitrobot chamber. (C) Assembled vitrification container set. (D) A grid plunged into liquid ethane on the Vitrobot.
  b. Attach new circular blotting paper to both blotting pads inside the Vitrobot chamber, with concave side of the paper’s inner rim facing the pad and close the chamber (Figure 9B).
  c. Turn on the Vitrobot.
  d. Set humidity to 100% and select ‘ON’ setting.
  e. Set temperature to 22°C.
  f. Inside a fume hood, assemble the vitrification container set that consists of a liquid nitrogen base container, a brass ethane cup, a grid-box holder, a spider, and an anti-contamination ring (Figure 9C). The anti-contamination ring should be placed with ridges facing up.
  g. Fill up the vitrification container set with liquid nitrogen (LN2), including the ethane cup, and keep the LN2 level in the base container.
  h. Wait until the liquid nitrogen evaporates completely from the ethane cup.
    ***Note:*** The ethane cup must be cold enough for ethane to liquify (its boiling temperature at atmospheric pressure is −89°C), but not too cold to solidify (melting temperature at atmospheric pressure: −183°C). To keep the cup cold, make sure that the spider that connects the LN2 bath with the ethane cup sits stably on the edge of the cup. If ethane comes into contact with liquid nitrogen, ethane will solidify almost instantaneously.
  i. Fill up the ethane brass cup with ethane.
    ***Note:*** Take care when filling up the brass cup with ethane. Rapid heat transfer that occurs when ethane is accidentally dropped into liquid nitrogen causes rapid evaporation of ethane and pressure buildup. Always wear eye protection gear when handling cryogenic substances. Ethane is highly flammable, and liquid ethane can cause frostbite on contact with unprotected skin.
  j. When needed, refill the liquid nitrogen base container from behind the anti-contamination ring, so that liquid nitrogen does not come into contact with liquid ethane.
  k. Place grid-boxes into the grid-box holder, just below the liquid nitrogen level.
  l. When the surface of liquid ethane turns slightly opaque, remove the spider and transfer the vitrification container set to the Vitrobot.
  m. For applying the sample onto grids freshly covered with graphene oxide use the following parameters.
    i. Blot Force on the Vitrobot set to 0.
    ii. Wait Time on the Vitrobot set to 60 s.
    iii. Blot Time in between applications on the Vitrobot set to 0.5 s.
    iv. Final Blot Time on the Vitrobot set to 4.0 or 4.5 s.
      ***Note:*** Values for Wait Time and for the final Blot Time can vary depending on the Vitrobot instrument used.
  n. For each of the three sequential sample applications, dispense 4 μL of sample (from step 14.m) onto the same side of the grid that was glow-discharged and coated with GO.
  o. After plunge freezing into liquid ethane (Figure 9D), transfer the grid into liquid nitrogen and store in a grid box until the day of the cryo-EM imaging session.
    ***Note:*** In our experience, the particle density observed in negative stain EM is similar to the one observed on GO-coated cryo-EM grids. Thus, based on estimates from UA-stained grids, the number of applications required to obtain suitable particle density in cryo-EM grids can be established. When plunge freezing several grids, take care to keep the LN2 level constant in the base container.

## Expected outcomes

### *Xenopus laevis* demembranated sperm nuclei

**Figure 10:**
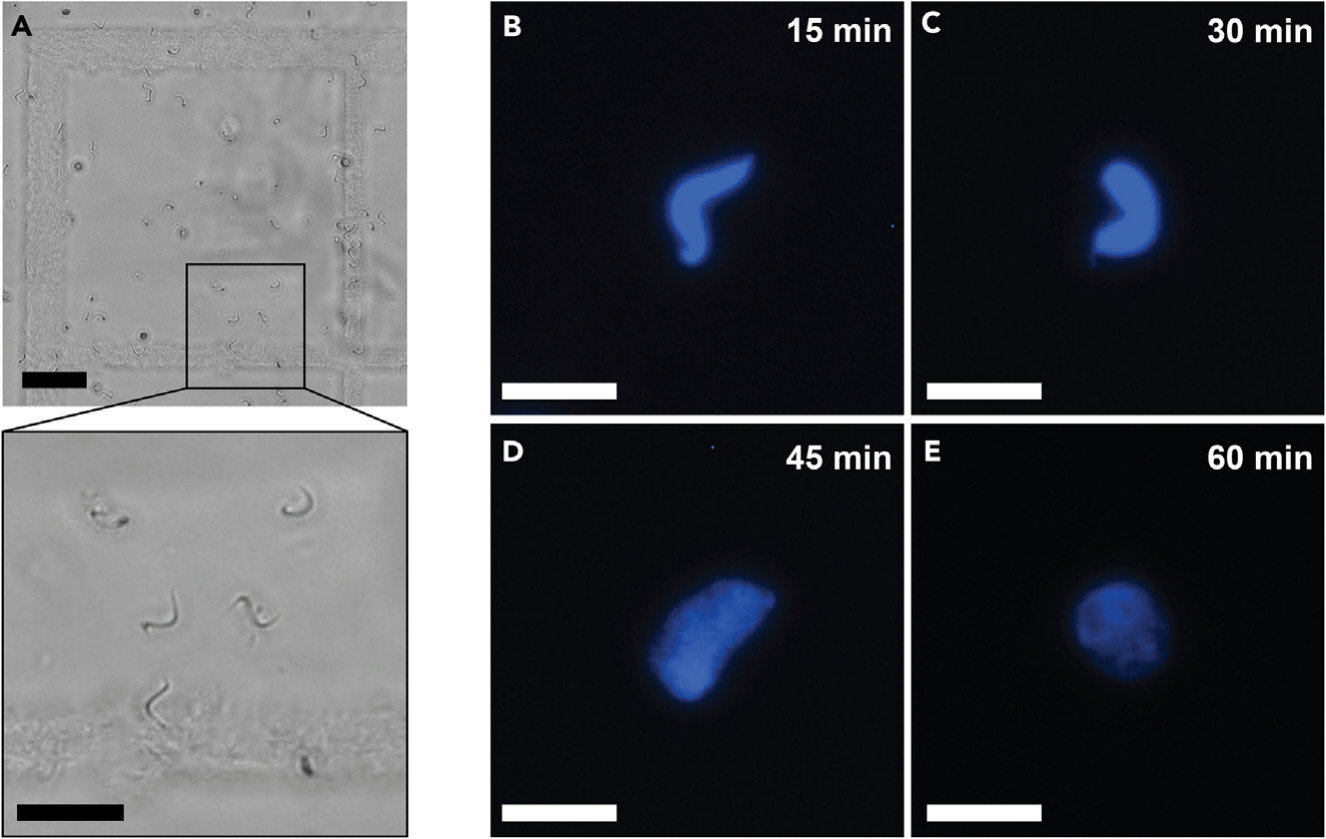
Quality evaluation of demembranated sperm nuclei (A) Sperm count with hemocytometer in bright field with 20x objective. Scale bar 50 μm, inset 25 μm. (B–E) Fluorescence images of nuclear formation and development. The reaction started when sperm nuclei are added to egg extract released in interphase (0 min), and was followed over 60 min at indicated times: 15 min (B), 30 min (C), 45 min (D) and 60 min (E). Sample stained with Hoechst 33258 (5 μM final concentration), scale bar 20 μm.

**Figure 11:**
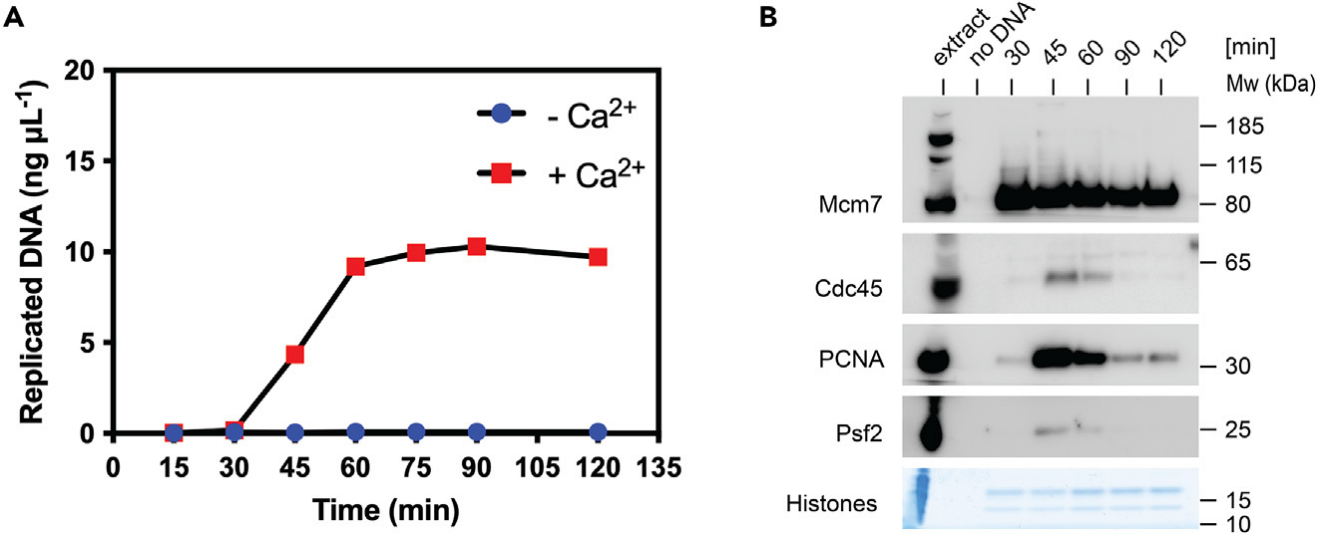
Quality evaluation of *Xenopus* egg extract (A) DNA synthesis was assayed via α^32^P-dATP incorporation detection. (B) Time course of DNA replication reaction with chromatin associated factors. The reaction was assembled with and without CaCl_2_. Chromatin was isolated at specific time points during DNA replication, separated via SDS–PAGE and immunoblotted with antibodies against main replication factors, while the lower portion of gel was stained with Coomassie blue to identify histones.

The sperm may be S-shaped or circular, depending on the angle they are observed, and preparations typically contain 1%–5% somatic nuclei. The count is repeated 4 times to ensure statistical accuracy (Figure 10A). The mean number is calculated giving double weight to somatic nuclei because these are diploid. A typical yield is 33,000,000–66,000,000 haploid nuclei corresponding to 100–200 μg DNA per testis. This is calculated considering that the *Xenopus* haploid genome is of ∼3 pg DNA. Demembranated sperm nuclei (DNA) are generally used with LSS at a final concentration of 10–20 ng/μL (Figure 11A). Precise DNA stock concentration is also determined via titration into LSS and measuring the level of DNA synthesized via TCA replication assay.^7^

### *Xenopus laevis* Low-Speed Supernatant Egg Extract

Prepared egg extract is tested for its ability to synthesize nascent DNA. Egg extract is supplemented with demembranated sperm and radioactive nucleotide, which is incorporated into newly synthesized DNA during the replication reaction. Measurement of α^32^P-dATP incorporation into DNA provides a good indication of replication capabilities and replication kinetics of produced extract (Figure 11A). Meanwhile, analyzing the time course of the DNA replication reaction through western blotting of the isolated chromatin fraction offers a robust approach to examine the proteins binding to chromatin at specific intervals during the replication process (Figure 11B). Both of these assays are very well described in Gillespie, Gambus Methods 2012 and we perform them in the same way as described.^7^

### Analysis of purified replisomes

**Figure 12:**
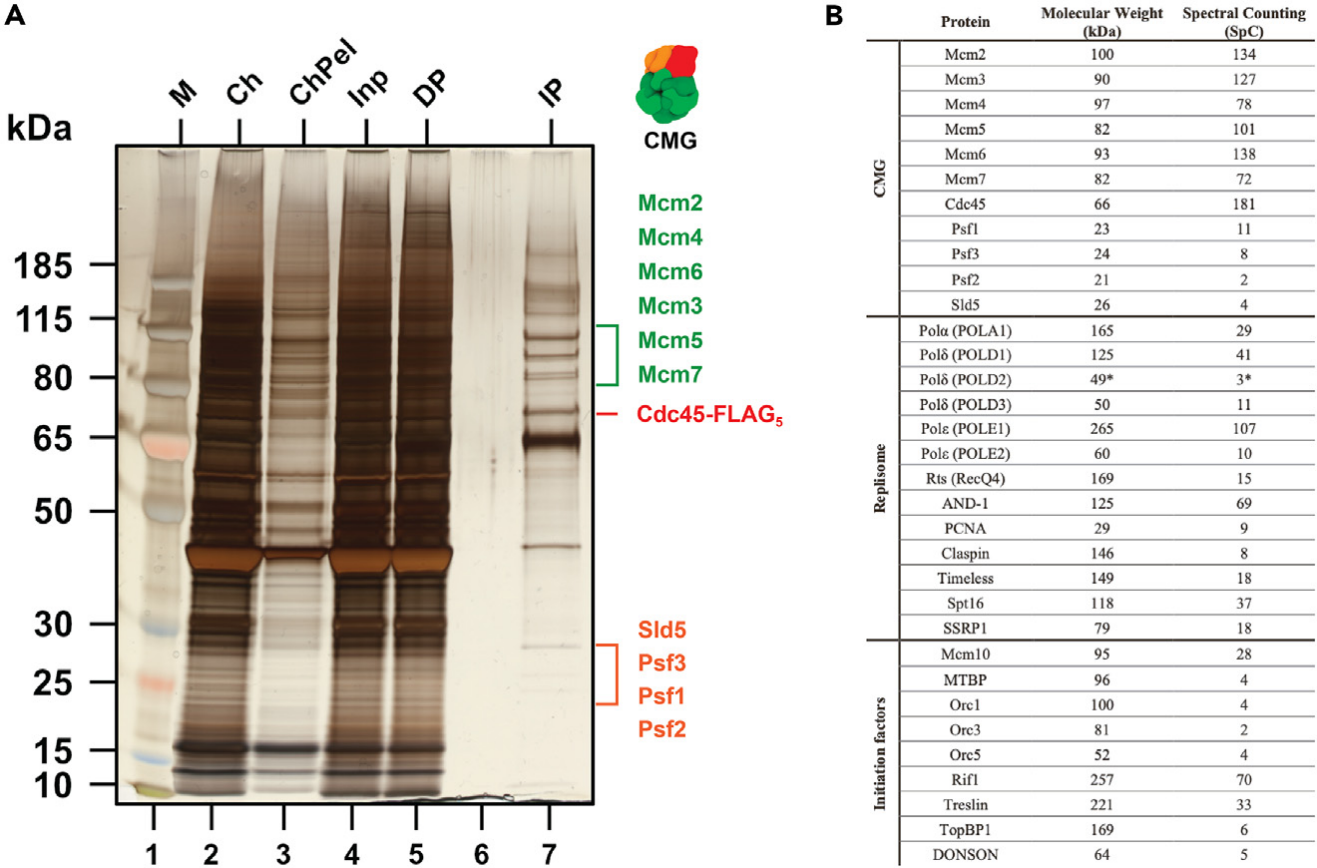
Assessment of purified replisomes after IP (A) Silver-stained gel of the large-scale FLAG-IP samples with CMG components highlighted. Adapted from.^1^ (B) CMG and other co-immunoprecipitated proteins from the IP sample, detected by mass spectrometry. Selected replication factors are presented with molecular weight and total spectral count. Full data available on PRIDE. (∗*Xenopus tropicalis* proteins). Adapted from.^1^

SDS-PAGE and silver staining can be used to assess the quality of the purified replisomes before proceeding with negative staining, plunge freezing and cryo-EM (Figure 12Α). Moreover, the same IP sample can be used for verification of complex components co-immunoprecipitated in the sample by SDS-PAGE and western blotting. Alternatively, composition of the final sample can be analyzed by mass spectrometry (MS) of either excised bands or a whole mixture (sample from step 14.m) as shown in Figure 12B.

**Figure 13:**
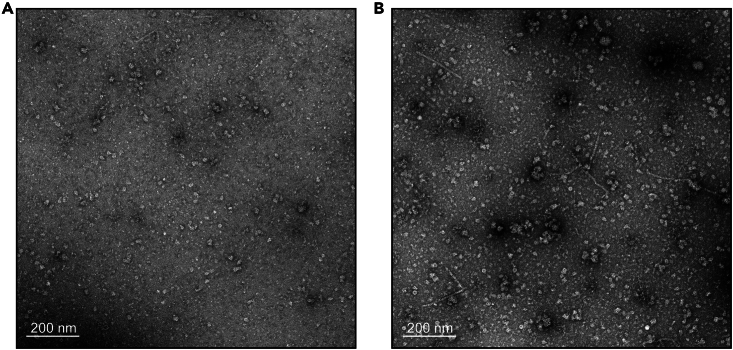
Multiple sample applications on a grid (A) Negative stain EM micrograph after single sample application on the grid. (B) Negative stain EM micrograph after three consecutive sample applications on the grid.

Direct analysis with NS-EM is used for assessing the particle quality of the sample (Figure 13).

## Limitations

The presented protocol utilizes release of protein complexes from chromatin through digestion of DNA with a DNase (Benzonase). The consequence of this approach is that there is no DNA associated with the protein complexes isolated. To visualize protein complexes interacting with DNA, a different approach to solubilize chromatin associated proteins is necessary. One could envisage utilization of restriction enzymes, or fragmenting nucleases such as the micrococcal nuclease.

The proposed protocol utilizes recombinant FLAG-tagged protein added to the extract to compete with the endogenous protein, incorporate into protein complexes and allow for their affinity purification and elution in native conditions. This approach requires prior purification of the tagged protein of interest, which may be challenging. To avoid this step, one can envisage the possibility to use antibodies raised against a peptide from a protein of interest. Such antibodies could be used to purify the complex of interest, while their interaction could be reversed with high concentration of the peptide they were raised against, leading to complex elution (akin FLAG-peptide elution).

The presented protocol is efficient for isolation and visualization of CMG complexes, which are stable in our hands. The analysis of other replisome components interacting with CMG helicases is more challenging. The CMG complexes containing additional densities on their peripheries are less abundant and heterogeneous. This could be improved by changing buffer and chromatin release conditions, while taking into account that the larger the complexes, the less soluble they are. As mentioned above, the conditions used here are a compromise between the complex size and solubility. Finally, one can envisage that gentle crosslinking could help to stimulate complex shape and composition retainment throughout the procedure.

## Troubleshooting

### Problem 1

In cases where the sperm preparation exhibits notable contamination from erythrocytes, identifiable as a red fraction settling at the pellet’s bottom (Figure 5Bvii) (related to step 4.g).

### Potential solution

These cells can be burst through resuspension and transfer of the sperm into another tube, and the addition of EDTA to 5 mM. The recovered sperm should undergo another round of centrifugation, discarding the remaining pelleted blood cells.

### Problem 2

Chromatin should be present at the bottom of the tube in the form of a white pellet. Sometimes filaments of chromatin are suspended in ANIB-sucrose and not pelleted (related to step 12.e).

### Potential solution

Centrifuge again for 5 min. Repeat until all chromatin is pelleted.

### Problem 3

Large complexes assembled on chromatin may not be soluble upon chromatin digestion (related to step 13).

### Potential solution

Increasing salt concentration at this point may break some of the interactions, reducing the size of complexes and retaining them in the soluble fraction. In our experience, CMG complexes are still stable at 300 mM KOAc salt concentration and are more soluble as they interact with fewer additional replisome components. The salt concentration used is therefore a compromise between the yield and the size of the retained large molecular size complex.

### Problem 4

After spinning a small brown/dark pellet should be visible. If a white pellet is present, that is chromatin, and it means that the digestion was partial (related to step 14.c).

### Potential solution

Sonicate for another 2 min (15 s ON – 15 s OFF). Repeat sonication until no white pellet is visible after step 14.c. Try to avoid long sonication and higher power to limit sample loss.

### Problem 5

If particle count per micrograph is low (related to step 16).

### Potential solution

The sample application step can be repeated several times (Figure 13). This can be done by performing points 16.f-g, although protein precipitation or damaging of the continuous carbon layer has been observed when 10 applications are exceeded. Longer incubation times using larger sample volumes can be also used to maximize particle count, however such procedure should be conducted in a humidity chamber to prevent sample evaporation.

## Resource availability

### Lead contact

Further information and requests for resources and reagents should be directed to and will be fulfilled by the lead contact, Agnieszka Gambus (a.gambus@bham.ac.uk).

### Technical contact

Technical questions on executing this protocol should be directed to and will be answered by the technical contact, Paolo Passaretti (p.passaretti@bham.ac.uk,) and Milos A. Cvetkovic (milos.cvetkovic@crick.ac.uk).

### Materials availability

There are no newly generated materials associated with this protocol.

### Data and code availability

The protocol includes all datasets generated or analyzed during this study.
